# Editorial: Transfusion Medicine and Blood

**DOI:** 10.3389/fmed.2018.00355

**Published:** 2019-01-08

**Authors:** Michel Prudent, Jean-Daniel Tissot, Stefano Fontana, Christoph Niederhauser

**Affiliations:** ^1^Interregional Blood Transfusion SRC, Épalinges, Switzerland; ^2^Faculté de Biologie et de Médecine, Université de Lausanne, Lausanne, Switzerland; ^3^Institute for Infectious Diseases, Faculty of Medicine, University of Bern, Bern, Switzerland

**Keywords:** blood, blood groups, blood products, donors, hematology, infectious markers, transfusion medicine

Transfusion medicine is in perpetual evolution and has faced several challenges since the seventeenth century. Nevertheless, it is a relatively new specialty based on an old and simple concept that is transferring blood from an individual to another one. The history of the discipline is quite rich, the first steps being associated with killing patients (1) (due to numerous reasons sometimes not necessarily related to transfusion) notably because of acute hemolytic reactions related to ABO incompatibilities (beyond xenotransfusion). Indeed, before 1900, the blood groups were unknown (2). Over the years, transfusion medicine evolved from a dangerous concept to a reality, and was really the first example of personalized medicine when transfusion was performed arm to arm: the donor and the recipient were in the same room, with a single doctor caring both individuals as well as the process (3). Furthermore, the blood transfused was a non-denaturized product: it was “true” blood, “pure” blood, without any process and storage lesions.

Nowadays, blood transfusion is the paradigm of both precision and personalized medicine: (i) blood or blood components are collected from donors with well characterized blood groups and/or HLA geno-phenotypes and or sometimes for particular (non)-reactivity with specific pathogens such as hepatitis E or cytomegalovirus, (ii) blood is separated in its different components that are red blood cells (RBCs), platelets, plasma, and other therapeutics. Whatever the “philosophic consideration,” more than 100 million of donations per year are performed worldwide and, even if at risk, transfused blood products save lives. Nevertheless, the pressure in terms of efficacy, safety, blood management, and cost constrains currently pushes a further evolution of transfusion medicine. Research in transfusion medicine is diverse, interdisciplinary, and regularly proposes innovation along the whole transfusion chain, involving every stage from the donors to the recipients. Each step (donors and blood collection, screening, preparation, storage, transfusion as well as post-transfusion survey) has been considered in order to improve safety, quality, and benefit to the patients. Some of these aspects are highlighted by the diversity of the published articles in this research topic and introduced below.

## Global and Individual Diversity

The diversity in transfusion medicine is real (beyond the blood groups), and this diversity is present at different levels. Socio-political and economical aspects of transfusion medicine are of primary importance when western countries participate to the development of blood collection and processing in lower to upper middle-income countries. It is the case in Eastern/Southern Mediterranean countries where surveys are currently ongoing to address diverse issues in transfusion medicine, as reviewed by Haddad et al. The diversity is also dependent on the donors (genetically or not), on the patients, and on the processing and storage, where differences in blood component quality were reported. Garraud and Tissot discussed these differences and similarities, and the potential consequences in transfusion.

## Safety and Infectious Disease Agents

Safety of blood transfusion is achieved at different steps of the chain. The first link is the donor questionnaire based on compliance where both donor and patient health are considered. The questionnaire and the interview are the results of several adjustments and efficient analyzes (as deeply investigated by Gillet and Neijens), where nurses and other health professional play a central role.

The next link is blood testing for infectious disease markers. The first blood donor screening test for an infectious disease agent to be routinely introduced was implemented in the early 1940s to detect a *Treponema pallidum* infection. Until the discovery and characterization of the Australia antigen (HBsAg) in 1968, no further tests were implemented. Up to the early 1990s many blood products were contaminated with HIV, HBV, or HCV, which consequently resulted in many transfusion-related infections. Following the AIDS scandal during which thousands of blood product recipients were infected, there has been a vast increase in specific and elaborated measures to prevent such dramatic infectious disease transmissions. These include the introduction of a sophisticated donor questionnaire, a rigorous disinfection of the skin associated with the elimination of the first few milliliters of the collection in order to decrease to load of skin-related bacterial contamination, the leucodepletion as a toll to eliminate intracellular microbes—beside the reduction of the immunogenicity and of the storage lesions of blood components, highly sensitive and specific serological tests and finally new test technologies such as nucleic acid testing (NAT) and pathogen reduction (PR). All these measures have greatly increased the safety of blood products in relation to the transmission of pathogens. In the last decade there has been a new focus on the so-called (re)-emerging infectious agents, such as West-Nile virus, Chikungunya virus, Dengue virus, and Zika virus. The interest in these diseases led to the introduction of new measures to prepare the blood donation community for the potential emergence of these threats. The current situation in the Latin American continent is described by Levi. Particularly in Europe the viral agents Parvovirus B19, Cytomegalovirus (CMV) and more recently Hepatitis E virus (HEV) have become an important issue. Juhl and Hennig and Dreier et al. show how Parvovirus B19 and HEV, respectively, are relevant in the safety of blood components. Despite the measures described above, there are still several issues which need to be addressed. For instance, it is often not clear whether a pathogen is really transmitted by blood products. If the agent is shown to be transmitted, then the question of the infectious dose needs to be addressed. Both, the transmission and the infectious dose, are essential issues which must be evaluated prior to the design of a specified test strategy. Furthermore, the importance of the virus load, as exemplified for HBV, is addressed by Candotti and Laperche. PR has recently become standard practices in many countries either for platelet concentrates and/or plasma resulting in safer blood products. It remains to be seen whether PR of whole blood and/or the automation of the procedure might, in the future, increase the efficiency. With the progressive introduction of various PR technologies, there is place for discussions how these technologies will either complement or replace the existing screening strategies. Seltsam reviewed in a very informative manner the recent developments in the PR of blood products completed by a comprehensive description of the molecular impact in platelets by Schubert et al. Particularly the possible implementation of an automated PR system of whole blood will lead to debates whether the current highly sensitive NAT systems required or whether less sensitive pool NAT systems will be sufficient. It is also possible to envisage an approach by using multiplexed viruses detection in one NAT reaction in order to save costs.

Ultimately it has to be clarified how, when and why new measures should be introduced. Appropriate decisions must be made together with all the various stakeholders involved, based not only on safety points but also on clear cost-benefit considerations. A well-adjusted safety policy will not be easy to define and certainly will not satisfy everyone involved in the global process. The balance between maximal or optimal safety and the high costs are relevant topics: the subject is elegantly discussed by Zaaijer in his open minded review.

## Understanding the Blood Products Quality

PR systems (as stated above) have recently become standard in many countries for platelet concentrates and plasma resulting in safer blood products (Seltsam; Schubert et al.). This innovation is a typical example where the processing impacts several biological functions of blood products. In-depth investigations of RBCs, platelet and plasma components have been carried out during the last decades. For instance, the impact of PR on platelets (reviewed by Schubert et al.) illustrates the role and the advantage of these investigations in understanding the preparation of blood for transfusion. The platelets thus produced exhibit different properties (we meet once again the diversity) that might be particularly relevant for recipients. Another interesting aspect of platelets physiology was pointed out by Handtke et al. They reviewed the relevance of cell subpopulations for transfusion which is of importance knowing that the method of collection and processing might enrich or deplete specific phenotypes. The next cutting-edge innovation is clearly the *in vitro* production of platelets. This field of investigation [as it has been achieved a few years ago for RBCs (4)] is clearly moving forward and Strassel et al. reviewed the advancements and challenges in this promising facet of transfusion medicine that could deeply transform the whole blood chain supply.

RBCs have been also deeply investigated during the last decades and the related articles illustrate here the achievements reached so far. Nemkov et al. have recently focused on metabolism of citrate in mature RBCs. Their interesting metabolomic investigation reports the consumption of citrate and other carboxylates in function of hypoxia. Using a similar approach, the same group in collaboration with Kriebardis and colleagues (Reisz et al.) highlighted the consequences on storage parameters when blood was collected from glucose 6-phosphate dehydrogenase (G6PD)-deficient donors. These investigations are not only relevant for better understanding of pathophysiological behaviors of RBCs but also for the investigation of storage lesions, which are the consequences of a cascade of events (5). At the other end of metabolism rerouting, cell morphology shifts from a discocyte to a spherocyte with an impact on deformability. Several tools are available to quantify these modifications and Roussel et al. described a simple and elegant method to assess volume and morphological parameters using a microfluidic chamber coupled to fluorescence exclusion without RBC pre-treatment. These approaches will be useful for as well the future developments of preservation strategies as the physiological understanding of RBC biology. The assessment of transfused RBC quality (knowing the deleterious effects of processing and storage) in clinical trials is required. The gold standard is obviously the 24-h post-transfusion recovery that is one of the specifications that has to be met in order to validate red cell concentrates (RCCs) for transfusion. Nevertheless, the different methods reviewed by Roussel et al. exhibited weaknesses and other markers of storability would be a plus in validation procedures and clinical trials.

## Patients

Last but not least, we should never forget that blood products are designed and processed for transfusion purposes. The role of RBCs in neurodegeneration was explored in a perspective paper by Bosman. The pathological context of the disease (here neuroacanthocytosis) and the RBC abnormality can affect brain oxygenation; the question on treatments that could reduce RBC defects is thus opened. The previously mentioned metabolomic approach on G6PD-deficient donors was also applied to assess the status of fresh frozen plasma (FFP) derived from these donors by Tzounakas et al. Their results highlighted different properties compared to standard FFP which might deserve the transfusion of plasma taking advantage of antioxidant and procoagulant activities. The transfusion of FFP has his pro and cons clinicians and recent studies plaid for beneficial effects of plasma. In this sense, Barelli and Alberio interestingly discussed the role of FFP in massive bleeding and its ability to preserve the endothelial glycocalyx structure and function. As for the platelet transfusion where their hemostatic role is obvious, Sut et al. pointed out in a detailed review the inflammatory potential of stored platelets and their involvement in adverse reactions.

Another important adverse transfusion event is alloimmunization resulting from the phenotypic disparities between donor and recipient. Repeated blood transfusions can result in the production of alloantibodies against one or more RBC antigens, which in turn complicate subsequent transfusions. Such events often lead to difficulties in providing a compatible RCC for a future transfusion, which can lead to an increased morbidity and mortality in transfusion-dependent patients (as in sickle cell anemia). Furthermore, due to the increasing heterogeneity of populations, the matching of the labile blood components for patients of different ethnicities is becoming increasingly awkward. van Sambeeck et al. reviewed the future approaches in blood screening and patient compatibility of the blood products to improve targeted donor recruitment strategies.

The thematic covered in this research topic on “Transfusion Medicine and Blood” is all but exhaustive: however, it represents recent advances and approaches along the transfusion chain, including specific preparations of blood-derived products such as the serum eye drop reviewed here by Drew et al. to treat dry eye syndrome. The clinical trials, the future of universal blood and all the therapeutics aspects of blood collection and cellular therapies were not included here but are part of our everyday life. The aims of all these researches are always to provide safe and adapted blood products for patients in a sustainable financial context, while keeping a suitable pool of health donors. It is interesting to point out that several papers in this collection consider the costs in their discussion since the margins are quite narrow to improve the whole chain.

In this context, research and development strategies are of primary importance to assist and shape the future of transfusion, and to convince stakeholders and other decision-makers. As in other scientific and medical fields, the economic pressure is important and it is difficult to grant research program. Moreover, the public funds are not always available for transfusion medicine and blood banks have to contribute to finance R&D by themself. Different organizations are in place worldwide partly supported by the academia (around two third of the authors here are employed in academia or university hospital) or 100% by blood banks. It is noteworthy that the investments are compulsory to shape the future and we cannot ignore the vast developments and innovation the R&D can initiate and support. Transfusion medicine has definitively been moving (even fighting against dogma since the 17th, dogma that still exist in different forms) and the science of blood transfusion is at the heart of medicine. Because, as already mentioned, transfusion medicine is both a personalized as well as a precision medicine.

## Author Contributions

All authors listed have made a substantial, direct and intellectual contribution to the work, and approved it for publication.

### Conflict of Interest Statement

The authors declare that the research was conducted in the absence of any commercial or financial relationships that could be construed as a potential conflict of interest.
